# Dental Items—Soft vs. Adhesive Gold Foils

**Published:** 1871-12

**Authors:** 


### EDITORIAL DEPARTMENT.

*Dental Items—Soft vs. Adhesive Gold Foils.*—Prof. C. W. Wright, of the Ohio Dental College, in an address published in the "Dental Register," advances opinions on the filling of teeth, which, as he remarks, may risk his reputation as an operative dentist. All will give him credit for candor, however much they differ with him in his method. He closes his address as follows:

"Because adhesive gold is good and valuable in certain cases, is no reason why we should make a large proportion of our operations *capital*, when they would be *minor* if we employed soft foils.

At the meeting of the American Dental Association, at Nashville, Dr. Forbes excused himself for not being able to make himself heard, on the ground of having lost his teeth by "first-class dentistry." Thousands of mouths are in like condition. The man who takes three hours to fill a tooth, should receive, by a law in political economy, as much for that one filling, as he should get for three, who fills three teeth in three hours. The slow method, therefore, tends to increase the price of an operation, the price of a tooth. Is there any advantage, or any object to be gained in raising the charges for saving a tooth threefold? Not if we are philanthropists as well as physicians and surgeons of the mouth. Unless our philanthrophy embraces only the dentist.

The greatest good to the greatest number is a good rule in operative dentistry. A great many men who have come into the profession within ten years, in our State, have never seen a soft foil filling, and do not know the advantages. To them I would say, try it in your laboratory, try it in simple crown cavities, learn the principle and test it for yourselves. Prepare your cavity without retaining pits—make cylinders, block or pellets a little longer than the cavity is deep. Set them in the cavity and apply lateral pressure against the walls of your cavity; wedge them in with a key block or cylinder—consolidate the surface with the burnisher, and finish. Or stuff your cavity full of gold, solidly against the walls in any way most convenient to yourself and patient, and the way you can do it quickest and best. Do this well. You can conserve teeth, avoid unnecessary pain, and fill twenty teeth per day—if the cavities are in favorable position—when you become expert. Reserve your adhesive foil for special purposes."

